# Retracted: Glycolipid Metabolism Disorder in the Liver of Obese Mice Is Improved by TUDCA via the Restoration of Defective Hepatic Autophagy

**DOI:** 10.1155/2020/2138532

**Published:** 2020-11-28

**Authors:** International Journal of Endocrinology

The article “Glycolipid Metabolism Disorder in the Liver of Obese Mice Is Improved by TUDCA via the Restoration of Defective Hepatic Autophagy” [1] has been retracted as it was found to contain duplicated figures.

As raised on PubPeer [2], there are concerns with Figures 2, 4, and 5. Details of the concerns are as follows:

- In Figure 2(g), the lanes for IL-6 are similar to the lanes in Figure 4(e) for p-eIF2alpha;
- In Figure 4(g), the GAPDH bands have nine lanes; however, the other samples have eight lanes;
- Figure 4(c) ND/Veh overlaps on the left-hand side with the right-hand side of Figure 5(c) Ad-GFP;
- In Figures 4(d), 5(c), and 5(e), several bands are duplicated: the left-hand three bands in Figure 5(e) eIF2alpha are highly similar to the right-hand three bands in Figure 5(g) Akt, bands 2–4 in Figure 4(e) IR when mirrored horizontally, and the right-hand three bands in Figure 5(g) IR when flipped vertically. Lane 5 in Figure 5(g) Akt is also highly similar to lane 5 in Figure 5(g) IR when flipped vertically. The right-hand seven bands in Figure 4(e) IR are highly similar to the right-hand seven bands in Figure 5(g) IR when flipped.

We asked the authors to explain this and provide the underlying images and raw data for all the figures. They said that the GAPDH bands in Figure 4, ND/Veh in Figure 4, and Ad-GFP in Figure 5 are all control samples and the photos might have been mistakenly selected but would not affect the conclusion of the article, and that the other bands were not duplicated. Given the extent of the issues identified, the editorial board recommended retracting the article. The authors do not agree with retraction.
